# The State and the Doctor

**Published:** 1906-03

**Authors:** Clair McKelway

**Affiliations:** Vice-chancellor of the University of the State of New York


### SPECIAL ARTICLE.

### The State and the Doctor.

By ST. CLAIR McKELWAY, LL. D.
Vice-chancellor of the University of the State of New York.

[*Albany Medical Annals.*]

I congratulate you on the attainment by the society of an age so respected and so long. I especially congratulate you on the condition of reunion and of fellowship which has been made finally effective and which renders this anniversary significant of far more than the mere age of your organisation which it attests.

There is, perhaps, a propriety in my addressing you. What you think of yourselves can be assumed. The effect of culture, character, competition and contention on a profession from which there is no appeal except to God and to the undertaker can be imagined—and is evident.

What an outsider thinks of you must be surmised. Officially I am an outsider. Sympathetically and by your kind indulgence I am likewise an insider. I often addressed this organisation before any line of demarcation was drawn in it. I subsequently addressed, with polar impartiality, not only the organisation which annually meets here, but also your temporarily separated brethren who met in Manhattan, which is sometimes incorrectly called New York. I invariably espoused the cause of the State Society before the State association. I did not hesitate to espouse the cause of the State association before this society. Some of your society would rather be kissed than cuffed, but while each of you insisted upon being right, the fact that your lines have been reformed on the basis of the old fellowship shows that the final right has been ascertained, and that lots need not be cast nor disputation multiplied as to which of the two was the more or less right—or wrong—at the time of separation.

I shall hold no such inquest. I shall neither suggest nor provoke taunts. The best way to agree, is to agree; the best way to maintain agreement is not to review or to revive misunderstandings which have been composed.

I am free to say that all doctors who meet here, and all laymen rejoice that the causes of separation are submerged in the fact of the reunion itself. The casuists might study these causes. The study might furnish a text or a pretext for vivisection. The process would be more industrious than benign. The conclusion would be more dogmatic than educational. It is enough — and it is gratifying — for us here and now to know that where there were two there is now only one society, and that for the long future as during the long past—prior to the separation—the physicians and surgeons of the State of New York represented in this society are and will be one.

OFFICERS ALWAYS REPRESENTATIVE MEN.

Before proceeding to any debatable topics and before indulging in any polemical suggestions, permit me affectionately to recall the pleasures of our long fellowship. Your officers now are representative men. Your officers in the past were representative men, both in citizenship and in medical science. Who here can forget the refined presence, the simple nature, the profound learning and the steady principle of Dr. Hun? All here will remember the subtle wisdom, the tactful diplomacy, the strong character and the hypodermic personality of Dr. Gray, the great alienist. None of us can have failed to mourn the recent loss of Dr. Didama, who, full of years and of honors from his profession and from his fellow-men, lately fell asleep in his home city, which regarded him as her most venerated son.

None of us here, going further back, can ever forget, while memory holds a seat and love a place in our hearts, Jacob S. Mosher, of this capital. He was a wit among scientists and a scientist among wits, and as a friend, a companion, a helper of his fellowmen, the memory of him is blessed. Nor is Wey, of Elmira, or Moore, of Rochester, or many a former officer of this society, lost to the mind. Their characteristics are cherished, their influence is still pervasive, and they can never be forgotten in the gatherings of their brethren.

I shall not suggest many names from my end of the State. They are well known to you, and hardly need suggestion.

Some of them were among my companions, and to them I cannot without emotion refer—they were my dear friends, and they were yours. We all know that Flint and Sims and Hutchison and Delafield and Mitchell and Crane and Chapman left upon the lives of their colleagues or of their pupils an influence which those pupils will, in turn, transmit to their successors, in coming generations. The light passes from hand to hand. The light is never put out. It is inextinguishable; it is immortal.

### THE ACCOMPLISHED REUNION.

I do not know under what conditions you have accomplished reunion. I feel sure the conditions were candidly canvassed and clearly understood. I can recall with distinctness the period of your separation and I, perhaps, could technically hint at the questions which led to it. I know they were intense questions. I know that the differences which they aroused were sincere. I know that the severances which they caused were acute and were grievous. I am sure that the respect which each of the former societies had for the other was unimpaired. We will do well to remember that those who went off from us did so without bitterness, and with regret. They will do well to believe that those who stayed by and held the fort felt that they should do so in order to be faithful to those with whom they acted and to the obligations of service which they had laid upon the State and the State on them. But we are here together again to-day and we are here to face, not the past with analysis or with acrimony, but the future with hope and confidence.

Many things have occurred. The average estimate of life has been lengthened. The list of diseases regarded as incurable has been made smaller. The percentage of mortality from difficult diseases has been greatly lowered. So large is this reduction that the revenues of your profession must have been perceptibly impaired, except in favored instances. Patients are fewer in number. Diseases are shortened in duration. The area of your lucrative practise and the period of complaints which make your practise lucrative have both become less. You may yet have to imitate the wise men of your calling in the East. You may have to charge, not for making persons well, but for keeping them well. Your income may have to be conditioned on the prevalence of health and may cease to depend upon your restoration of ill patients to health. The wise book says: "Those who are whole need not a physician." The time may come here, as it has come in the older, and, as it seems to us, the less cultivated portion of the world, when the physician will need those who are well and whom he keeps well, to be the measure of his income and the warrant of the confidence or of the competence he would command. Fancy could multiply comedies out of what may seem to you to be a paradox, but the paradox of one age may become the acknowledged principle of another. It often has.

FIELD OF THE SPECIALIST ENLARGED.

Why life has been measured with more profit to the insurance companies than to the policy holders has become evident. Complaint against that inequality is widespread. But why the doctor who keeps us well should not be so well regarded as the doctor who makes us well, after we have made ourselves ill, might be sincerely, instead of cynically or clinically, asked. This is not so absurd as it may seem. Your profession has divided the patient into compartments. Your profession has subdivided its members into specialists. The general practitioner still obtains, and many of them, I am glad to say, can be seen here tonight. But he is accustomed more and more to call the specialist into consultation with him. He can bring to the specialist a thorough account of the personality, the environment and the life and habits of the patient. The specialist can bring to him an accurate knowledge either of the complaint from which the patient suffers or of that organ of the patient which is particularly affected by such complaint. I recall Disraeli's definition of a medical consultation. "It is," he says in Lothair, "an occasion on which the consulting physician indorses the policy of the superintending practitioner—and changes the treatment."

I shall seek to explore none of the mysteries of your calling and to turn up none of the secrets of your prison house. If "I could a tale unfold whose lightest word" would accomplish all the dire results which Hamlet, in his soliloquy, both predicted and feared, I would not. But I would have you bear in mind that you must seriously consider whether, by your very skill, you may not be undermining your own practice and impairing your own revenue. Of course, those nations which we flippantly call "heathen" are indifferent to the ailing, impatient with the aged and almost hostile toward the dying. So were the Greeks. So were the Romans. So is not modern civilisation. There must be harmony between due sensibility to suffering and the pride and power commanded, and demanded, by health. The new dispensation must ameliorate the hardness of the old; but must borrow from the old some of the value which that old placed on health, for the sake of health, and on the usefulness of health itself to the age and to the State. And your own profession must realize that the State—as that political expression is understood by this generation—takes, not your quarrels, not your differences, not your divisions, not your scholastic distinctions into account, but your patients, both as citizens and as sovereigns. The State recognised different schools of medicine before they recognised one another. The State long waited for now legally recognised schools to agree with one another, and when it found that they did not, could not or would not do so, then the State came in and did its own work of recognition. That accomplished more than was at first realised.

THE STATE-MADE DOCTOR.

It shifted the center of power from over the heads of doctors to the State government. A doctor thereafter became a State-made product. He remained a medical college pupil, but as a doctor he became, if not a product, then at least the creature, of the State. New York well nigh led off in this. Other States have since assumed control of doctor-making in the final stage. The tendency among many States is indifferent to the ramifications of groups of your calling whether they flock together or, in the language of Dundreary, "flock separately." As already said, the State did not willingly or suddenly or violently assume control of the making or of the recognition of doctors. The State gave them plenty of time and plenty of hints to do that themselves. Those opportunities were not availed of. The schools which the State recognised, as organised facts, then collectively became the subject of State consideration. The State established for all intending surgeons and doctors a uniform degree of primary instruction. The State saw to it that the different schools, as nearly as could be, established a uniform degree of direct medical instruction. That set the present system going in this commonwealth. On the whole, it has gone on very well. It is not ideal, but it is practical. It is also progressive. It carries in it for medicine a reasonable assurance against ignorance and for patients against quackery. If, however, patients insist on preferring quackery, they have a constitutional right to do so, but they must prefer it openly. It cannot be palmed on them covertly, under State auspices. This is a great gain.

The question is practically settling itself. It was supported, and it was opposed. There were arguments for it, and there were protests against it. These will long continue. Some things, however, must be regarded as established. The State is and will remain the guarantor of doctors. Practitioners or colleges are their teachers, and will remain so, but the State will be—to speak practically—the final doctor-maker, and its stamp must be the last affixed. Those who receive its stamp will be the only ones. "None others genuine" will be the excluding language, or fact.

The present issue is not shall this be undone. It will never be undone. The pressing question is, shall more of it be done than is already done? Here is where the State must again come in. Without forecasting its action, I think one can tell where the State will take its stand. It is where we should all wish the State to take its stand, and having taken it, to hold it. Nearly every year the State is urged to recognise some new division or branch of alleged medical theory or practice, in addition to those already recogonised. Whether the State will do that or not is not for you nor for me to determine. But it is for you and for me, as citizens of the State, to consider, and by our consideration to help this State to a right conclusion. The State should not and never will lower the standard of primary medical education. It should not appreciably lower the standard of specific and final medical instruction for those for whom it maintains it.

The State should insist upon the literary and clinical requirement of that instruction, and upon the final examination of the steps of that instruction, under State auspices, within the subjects to which each school is limited and addicted.

Incidentally that final examination is, to a degree, under the supervision of the Regents. The Regents do not name the examiners. They make selections of them from among the names submitted to them by the three medical divisions recognised by State law. Nor do the Regents propound the questions. They are propounded by the representatives of the different schools named to the Regents. The examinations are rated in the usual mathematical and impersonal way, which is admitted by all to be practically just.

FUNCTION OF THE BOARD OF REGENTS.

The pleas made to the Legislature and through the press to the Regents and the politicians for additions to the list of medical divisions to be recognised by the State are natural and are pathetic. The sincerity of these appeals is manifest and should be cheerfully admitted, but they should really be addressed to the State. They cannot properly be addressed to the Board of Regents. That board simply receives orders from the Legislature. That board has respected the orders it has thus received. That board is limited by those orders. That board never sought those orders; it never desired them, but it has never declined them. It has simply obeyed them. The board, legally authorised and protected by the Constitution, is personally named by the Legislature. Its policy of awaiting the orders of the Legislature is alike loyal and logical. You, gentlemen, represent the senior and the more numerous and the more influential school of medical theory and practice. You are yourselves authorised to meet, before the committees of the Legislature, all applicants for further legislative recognition in the field of medicine. It is to the Legislature and not to the Regents, you should signalise your proverbial preference of peace to war and of harmony to discord.

You should leave to the Regents the execution of the will of the State. You should not expect of the Regents the retardation or the expansion of that will; but I can assure you that the Regents —as I have said—will favor the maintenance of existing elementary educational tests, for intending students of medicine as a preliminary condition to the commencement of their studies. Should new applicants seek to avoid or to lower these preliminary tests, their prayer to the State to suspend or to reduce or in any substantial degree to evade these tests should not, in our opinion, and I am sure should not, in your opinion, be granted, except on conditions to be clearly understood. Should new applicants meet preliminary tests, their claim to final State examination will remain for the Legislature and for the Governor to settle, and for the Regents, so far as they conscientiously can, to maintain a loyal regard to the ordered will of the State.

I have set forth these plain facts and have made these plain distinctions, for a plain reason. The Board of Regents, of which I still have, for a short time, the privilege to be a member and the presiding officer, has been urged to support the application to the Legislature of interests and of organisations which claim a medical recognition that the law does not at present allow or extend. My colleagues and myself have found life made more lively than we could wish by appeals, and are conscious that the appeals themselves have behind them a considerable body of sincere opinion and of earnest sentiment. We have been just as earnestly urged to repel this sentiment as to recognise it; just as earnestly urged to oppose it as to favor it.

THE PRIVILEGED AND THE UNPRIVILEGED.

The whole situation grows out of the State's assumption of the conditions to determine both initial and final educational tests in medicine and in surgery, in lieu of leaving, as in the past, the determination of them to the different and to the differing medical schools themselves, which were unable to agree upon them. It is natural for those benefited by present conditions to wish to retain them and to prevent others from sharing them with them. It is natural for those who would also share them, but who do not, to try to obtain them. The Board of Regents is thus beset both by the privileged and by the unprivileged. It is made very conscious of the existence of both and of its bombardment by both. We shall never presume the State will reduce its preliminary educational tests. As already frankly stated, we do not believe the State should, nor can we presume the State will, reduce its final professional educational tests. It should require every one to know the how and why of what he proposes to do and should then allow him medically to do nothing else under States' auspices.

Frankly, should the State do less, the Board of Regents could well ask the Legislature to relieve it from all the medical work delegated to it, and unasked by it. Like work, however, has been delegated to us in the case of other learned professions. The improbability that the State will lower its standard is as plain to us as the fact that the State should not do so.

But there can be no reason for supposing that, given an equal degree of preliminary knowledge and given an equal standard and an equal period of scientific education, the board will be inhospitable to any new claimants for medical consideration by the State, who ask for what is just and fair.

The career of organised medicine has been marked by many advances. It has been signalised by many enlargements. It has been modified by many classifications. These have taken effect upon medical and surgical study and practice. All these events justify the supposition that evolution has not been brought to a stop and that the end has not been written against any branch of scientific study or practice.

I know these questions have been threshed out before, but there is need to thresh them out again. That must probably be done this very winter. Legislators and school men have been urged with uncommon vigor to lower the standards, on the one hand, so as to let in the ill-prepared, or so to enforce the standards, on the other hand, as to make the present beneficiaries of them their exclusive possessors. On the one hand we have been asked to vulgarise the standards; on the other hand to monopolise them. The State has the power to do either. I confidently predict the State will do neither. No steps backward have been taken by the State in medical education. Steps forward were long too few and too slow. At certain times they were too many or too quick, but the average maintained, while it may be raised or made less inelastic, will never be reduced, and that average here is less than that of more boastful States.

### A GLIMPSE AT CO-EDUCATION.

There is a series of propositions which grow out of the general statements which I have advanced, but which have a relation to the subject we are directly considering. Institutions, in New York for instance, on private foundations, in a few instances, illustrate co-education. State institutions, in Western commonwealths, illustrate that and those States will not recede from it. Former generations of voters in newer States, for reasons of economy, introduced co-education. For reasons of progress and of justice, and no longer merely of economy, they have carried co-education from the primary clear through the university. They love the system. Their fathers and their mothers were educated under it. This generation, gratefully, loyally and proudly maintains it. Our own commonwealth has not yet provided the capstone of free university education, to crown the foundations of free primary and secondary education. But, right or wrong, the plea is spreading, that our own commonwealth should do so.

The commonwealth is richer than all private wealth. From this fact grows, and is growing, the belief that the State should freely bring within the reach of all its children everything that private wealth can bring within the reach of its beneficiaries. "The best is alone good enough for all." This condenses the policy of the Middle Western, Northwestern and the Pacific States. This perhaps prophecies the pregnant purpose of the awakening South. The belief is gaining that New York itself will come to the substantiality of this. Some of us may not live to see it. Some of us may make our lives bitter by the futile violence of unproductive activity against it. But it will come, and we will go, and the memory of us, when we are gone, will be sweet, in proportion as we shall have foreseen and welcomed the larger and the better day or the era will pass by, unhonored and unsung, if we carp, and we will die disobedient unto the Heavenly vision. The free provision of the highest education by the State is, I think, ultimately inevitable. Whether or not it should so be provided for the sexes in separate institutions, or co-educationally, is a detail. The detail is not important.

This has a relation to medical education. The State insisted on prescribing primary standards to its own schools and then monopolised the control of intermediate ones through its own boards. After doing both it reserved to itself the licensure of medical, legal, dental, pharmaceutical and other learned practitioners. Whatever it may then have contemplated or intended, it then created the demand it should eventually provide and control the institutions themselves, whose human product it vises, examines and licenses. I am not unaware of the amount of property involved in private ownership of scientific school foundations. Neither am I unaware of the cost of the maintenance or of the management of private foundations. Nor am I unaware of the very gradual process of public opinion and of public action. Generations will probably pass away before the State will completely control the education of the children, from the kindergarten through the university; before the State will teach and train as well as make doctors, just as it now finally examines them and exclusively commissions them. Nor should one for a moment conclude that there will be, or that I would advocate, the abolition of private foundations. There will be a division of higher learning between the State foundations and private foundations.

Such a division already obtains in commonwealths which have long conducted State universities. Nothing essential, however, will eventually be beyond the reach of the children of the commonwealth. at the hands of the comonwealth, which is now within the reach of the children who can command the benefits of private wealth. There will be no compulsion or confiscation, but there will be discrimination. The State must eventually put within reach of all what private endowment, or private munificence, now puts within the reach only of some.

### DEVELOPMENT THAT IS INEVITABLE.

My friends, whether we realise it or not, whether we foresaw it or not, this became inevitable when high schools. normal schools and normal colleges were established in the process of public education. The State slowly parted with the idea that it was bound to give only the minimum of education to its children. It slowly realised it was bound to place within their reach more than as much knowledge as would keep them out of jail, if they acted on it, and justify the putting of them in jail, if they refused to act upon it. The State slowly grew to the idea that it should raise the schooling of its children by its own hands; at its own cost; by its own teachers, from the minimum to a modicum of education. But when the people were providing the modicum of education, the certainty, at some time, they would provide the maximum of education became apparent. The State now says "No one shall be a doctor or a lawyer or a dentist or a pharmaceutist or an accountant until I shall have examined him and until he shall have been commissioned by me." When the State said that, with the approbation of the callings or of the professions therewith concerned, the State gave entrance to the idea that it might control, and that it might conduct, institutions of its own like unto those, from which are now sent up to the State graduates upon whom it stamps its own imprimature. Only those whom the State thus authorises and credentials can deal with human rights, with human life, and with what is affected by an intimate relation with human rights and with human life. There can be, and there ought to be, a degree of resistance to this, for resistance to this will be salutary. Such a resistance will of itself, be desirable to slow the process which should not be too fast. There is, and there will be, denial of this. The denial itself will be desirable, to bring out the salutary agitation which is the spiritual and intellectual and moral warrant or prelude to the necessary education. Protest against this will be desirable to open the way and to preview the steps necessary to the establishment of this. Protest will give to beaten objection itself the satisfaction of knowing that it had its day in court, of knowing that it was not brutally overridden by the impact of unreasoning, impatient and irresistable insistence.

WHAT THE STATE HAS DONE.

Much that I have before spoken to you in former years, and written about you in past times, can be quoted to the contrary of this. "When I was a child I thought as a child, I understood as a child; when I became a man I put away childish things." Since then I have seen the State in its cities establish kindergartens and high schools over phrenetic protests against both. I have seen the State establish normal schools and normal colleges against the contention that "To teach teachers to teach is as absurd as it would be to teach mothers to nurse or children to play." Well, the mothers of the overworked poor are now taught how to nurse; indeed, their children are even nursed for them amid clean and sweet surroundings, while the mothers are away at hard work in congested city centers. To-day the children of the slums are gathered at kindergartens or in city playgrounds. To-day they are tenderly taught even how to play, instead of leaving the instinct for play to the outcome of chance, amid conditions of confusion and of dirt and the barabarisms which combine to make for sin. He who took the little children in His arms and blessed them and said of them "Of such is the kingdom of Heaven" has touched city, county, State and national life, and the human heart, with His sublime spirit. He has made much of our life the almoner and exponent of His life to many of His little ones. Very significantly, the race of His Mother, in our free American cities, has been in advance of other races, to take its children in its arms and to bless them with the blessing of education, tenderness and training; putting their little feet on the paths of right endeavor and leading them tenderly over the steppes of elementary learning to the flowery plains of trained culture.

We cannot arrest this manifest tendency if we would. Eventually the State will be as bound to complete and to perfect what it begins, as the moral and spiritual law itself, in pursuance of which the State acts, is bound imperceptibly, invisibly but omnipotently to have its way in the heart of things, and in the hearts of men. This will not be a socialism that levels down. This will be the spiritual regnancy which levels up. Nor will the State be discouraged, should the facilities it must ultimately provide be at first availed of by a very few. A splendid hospital is a public pride and a public benefaction. It is not a failure, if it be not full of patients. It is not a failure if it does not "pay" in the things of the market; it is an expression of the things of the spirit; it makes for health and happiness to the suffering; it attests the justice, the altruism and the love of the State for humanity at large.

THE CLAIM TO FREE EDUCATION.

The few who might go at the first in our commonwealth to such colleges or such universities—which would involve the teaching of medicine by the State—would not affect the duty of the State to provide for them, and the few at first who might attend would not long deter the children of the State from availing themselves of these privileges in larger numbers. As the State is richer than all its private wealth and as all its private wealth is protected by State laws, and bequeathed at all only by State permission, and as all State law is conditioned on State justice, and is self-maintained by State strength and by State conscience, so should, so will, State institutions of higher learning and of the highest learning be at least as good and as fine as any like institutions upon private foundation. The higher supply could at first be limited to the measure of the higher demand, but the demand would shortly increase and the supply would be correspondingly augmented. I have before other institutions in the past enlarged on the benign factor of privation as a stimulus to the soul of the poor, bent on getting learning, but I am now satisfied that the poor child in the rich city is entitled to free education to the limit which is at the command of those not poor.

This may cost a season of protest in colleges and hospitals, and may cost the State some reviling from both in the minds of those to whom private foundations are a form of wealth or income. That is natural; that must be allowed for; it should not be forgotten, however, that from the State or from its municipalities, private medical foundations already receive a large measure of public money. They undoubtedly deserve it. But the argument against State ownership and State conduct of hospitals and medical colleges could better be urged by others than by State beneficiaries in control of such more or less subsidised institutions. State institutions of this kind need not—as already said—displace private institutions of this kind. There are colleges and universities on private foundations in commonwealths maintaining State colleges and State universities. There could, there should, there will be hospitals and colleges upon private foundations, should this State establish others on its own foundations. The State could have relegated all higher education to private initiative and support. The State long did so. But refusing longer to do so, the State opened the way for itself as an educator from the foundations to the pinnacles.

GOVERNMENT INEXORABLY LOGICAL.

Government is inexorably logical. Government as large as that of New York State may be halted from expansion. It may be checked by temporary considerations. But it will not long be halted or long be checked. Government is the ultmate of public opinion. Public opinion in the end is expressed by government. The laws that cross that public opinion are changed. The constitutions that stand in its way are amended, or are interpreted, in line with it. There are few things more slow than the outcome of public opinion into law, within, around, over or through constitutions. The fathers—as if deliberately—sowed the path of progress or of change with obstacles so as to check the process of change itself. But change is certain, though slow. The present trend of progress is manifest. It is toward the doing for the people by the government of several vital things which government has heretofore been disposed to leave to private initiative or private combinations. The liberty thus accorded to private initiative or private combinations has been abused. The shores of our time are grimly lined with the wrecks of character and of manhood that could not survive the pressure of injury or live in the white light of impartial justice. Political parties to-day are desperately trying to evade the consequences of their own defaults. They are endeavoring to realign themselves around sham issues in mock contention. They are falling, in many quarters, to pieces, under the destructive strain of a systematic, desperate and panic-stricken insincerity. So the courtiers of King Canute in vain urged him to veto the incoming ocean. So did the shivering poltroon, pictured by the French artist, in the beginning urge the Creator "to conserve chaos." So did King George discard Pitt and Burke and lean on Lord North, only to lose his colonies and to make the bounds of freedom wider yet. In the light and under the force of steady pressure of this ethical time toward changed and better conditions, and for new and purer instrumentalities—a pressure which can be charged with dramatic displacements and the reversal of many long-established propositions—under the light and force of that pressure should be judged, and can be foreseen, the manifest destiny of the State to take much of the higher education, as it has already taken nearly all of the primary and secondary education, into its own hands. Complete medical education under State auspices may on these accounts be surely predicted, and what is of more importance may be safely advocated and gladly accelerated and welcomed.

